# Experiences of Caregivers as Clients of a Patient Navigation Program for Children and Youth with Complex Care Needs: A Qualitative Descriptive Study

**DOI:** 10.5334/ijic.5451

**Published:** 2020-11-10

**Authors:** Alison Luke, Kerrie E. Luck, Shelley Doucet

**Affiliations:** 1 Centre for Research in Integrated Care (CRIC), University of New Brunswick Saint John, New Brunswick, CA; 2 NaviCare/SoinsNavi, Saint John, New Brunswick, CA; 3 Department of Nursing and Health Sciences, University of New Brunswick, Saint John, New Brunswick, CA

**Keywords:** patient navigation, complex care, caregiver, qualitative description, integrated care

## Abstract

The number of Canadian children and youth with complex care needs has continued to rise, and their need for resources across all sectors can be extensive. Navigating the maze of resources and services can create confusion and impact how care is delivered and integrated. Patient navigators can help support and guide patients and caregivers through the healthcare system by matching their needs to appropriate resources with the aim to improve access and promote the integration of care. This qualitative study explored caregivers’ experiences caring for a child or youth with complex care needs, and their experiences and satisfaction as clients of a patient navigation centre. Participants included 22 clients from NaviCare/SoinsNavi, a patient navigation centre in Canada for children and youth with complex care needs and their families. Three main themes emerged: 1) caring for a child or youth with complex care needs, 2) navigating the system, and 3) the value of patient navigation. Findings suggest caregivers caring for a child or youth with complex care needs often feel overwhelmed, fearful, and alone; yet, patient navigation can be an innovative approach to support their needs through facilitating more convenient and integrated care, and improving access to education, supports, and resources.

## Introduction

Over the last few decades, the number of Canadian children and youth with complex care needs has continued to rise [1]. While this group still makes up only a small subset of the pediatric population, their need for resources and services across health, social, and education sectors can be extensive. Children and youth with complex care needs are defined by Brenner et al. as individuals with

“multidimensional health and social care needs in the presence of a recognized medical condition or where there is no unifying diagnosis. They are individual and contextualized, are continuing and dynamic, and are present across a range of settings, impacted by healthcare structure” [2, p.3223].

Caregivers of children and youth with complex care needs are often expected to become experts in managing care, as well as adjusting their lifestyle and environment to meet the child or youth’s needs [3]. These additional stressors and demands often result in neglected caregiver needs. Navigating the complex maze of resources and services can create confusion and can also impact how care is delivered and integrated. As a result, many caregivers and families feel overwhelmed and experience barriers to accessing the needed supports and resources they require to properly care for their child or youth with complex care needs. An integrated care approach is needed to support caregivers and families, which refers to a person-centred system approach that is achieved through the comprehensive delivery of quality services across the life-course to address specific multidimensional needs [4]. These services are ideally delivered by a team of providers working collaboratively across disciplines, settings, levels of care, and sectors.

Patient navigation programs are becoming recognized as an integrated care approach to support families in overcoming barriers and accessing resources and services for individuals with complex care needs. The first patient navigation program was developed and implemented in 1990 in Harlem, New York, by Dr. Harold Freeman [5]. As a patient-centred approach, patient navigation programs are increasingly being used across North America and abroad to support the timely movement of a client through a maze of services and programs across settings and sectors [5]. Thus, the goal of patient navigation is to help support and guide patients through the healthcare system by matching their needs to appropriate resources with the aim to improve access, and promote the integration of care [5,6,7].

A research-based patient navigation centre for children and youth 25 years of age or younger with complex care needs and their families was launched in a small semi-rural province in Canada in January of 2017. The aim of NaviCare/SoinsNavi is to help facilitate more convenient and integrated care to support the physical, mental, emotional, social, cultural, and spiritual needs of children, youth, and their families using a personalized family-centred approach. The centre employs two patient navigators, one a registered nurse and the second a lay navigator. The centre is unique compared to other patient navigation centres in that it is not disease or condition specific. A detailed description of NaviCare/SoinsNavi has been previously published [8].

Given that patient navigation programs are increasingly being used to help patients and their caregivers overcome barriers to accessing care, it is important to understand the experiences of clients using patient navigation services. Few studies have explored the experience of caregivers who use patient navigation services, particularly when caring for children or youth with complex care needs. Our aim, therefore, was to explore the experiences of caregivers of children and youth with complex care needs who have received services from NaviCare/SoinsNavi. Throughout this study, a caregiver is defined as a family member, friend, or guardian who provides the majority of day-to-day assistance and manages the wide array of care needs that confront the individual needing care [9,10]. The specific research questions of this study are as follows:

1. What are caregivers’ experiences caring for a child or youth with complex care needs?
2. What are caregivers’ experiences and satisfaction as clients of a patient navigation centre for children and youth with complex care needs?

## Methods

### Study Design and Ethics

The following study employed a qualitative descriptive design to gain an in-depth understanding of caregivers’ experiences caring for a child or youth with complex care needs, as well as their experiences and satisfaction as clients of a patient navigation centre for children and youth with complex care needs. Ethics approval was received from both the University of New Brunswick and Mount Allison University Research Ethics Boards. Informed consent was obtained from each participant prior to participation.

### Setting

Participants were recruited through NaviCare/SoinsNavi, a research-based patient navigation centre in a small Canadian province for children and youth with complex care needs. NaviCare/SoinsNavi offers free, bilingual navigation support to youth, caregivers, and care providers across the province. This study is part of a larger ongoing evaluation of NaviCare/SoinsNavi. Part of the development and implementation of this centre was establishing a Family Advisory Council (FAC). The seven members are either parents of children and youth with complex care needs or were themselves children or youth with complex care needs. The FAC advises all aspects of NaviCare/SoinsNavi, including the ongoing evaluation and research of the services provided.

### Sample

The population of interest was caregivers of children or youth up to age 25 with complex care needs who were clients of NaviCare/SoinsNavi. During the intake process with the patient navigator, all clients were asked if they would consent to being called by a member of the research team about a qualitative study. Clients who agreed were contacted by the lead author once their case was closed by the patient navigator. In total, 22 clients agreed to be interviewed for this study.

### Data Collection

Participants were interviewed individually by a member of the research team between July 2017 and July 2019. All interviews consisted of open-ended, semi-structured questions exploring experiences caring for a child or youth with complex care needs, as well as experiences and satisfaction as clients of NaviCare/SoinsNavi. Interviews were held either in the participant’s home, over the phone, or at a location chosen by the participant. Length of interviews ranged from 20 minutes to 2 hours.

### Analysis

All interviews were audio-recorded and transcribed verbatim. Once transcribed, the researcher employed Braun and Clarke’s [11] six stages of thematic analysis to pull out themes and sub-themes using NVivo 10 software. Rigor was achieved through independent consensus coding with two authors. An inductive approach was used when assigning codes to data. A code book was created employing an iterative process where interviews were revisited to ensure no new themes or sub-themes emerged from the data. Relationships between codes were determined with the use of concept mapping and further discussion with the research team. Data collection and analysis was completed once no new data significantly added to existing themes and sub-themes. This paper reports on themes related to the experiences of caregivers with children and youth with complex care needs after they have worked with a patient navigator employed by NaviCare/SoinsNavi.

## Results

Participant characteristics can be found in Table 1. In total, 22 caregiver NaviCare/SoinsNavi clients agreed to be interviewed. Participants were mostly mothers between the ages of 30 and 50 years, with a minimum of 4 years of university education. Employment status of participants was split between full time, part time, and currently not working. The most common reason for calling was to find resources and services.

**Table 1:** Participant Characteristics (n = 22).

	n

Age (n = 20)^a^	
20–29 years old	1
30–39 years old	9
40–49 years old	6
50–59 years old	1
60–69 years old	3
Gender	
male	3
female	19
Primary language spoken	
English	21
French	1
Highest level of education completed (n = 21)^a^	
four or more years of university	15
some university/college diploma	3
high school	1
some high school	2
Employment status	
full-time employment	7
part-time/casual/self-employed	6
currently not working	9
Gross family income (n = 20)^a^	
$19,999 or less	3
$20,000–39,999	2
$40,000–59,999	3
$60,000–$69,999	3
$70,000 or more	9
Marital relationship status (n = 21)^a^	
married/common law	14
separated	3
widowed	2
single	2
Relationship to the child	
mother	18
father	2
grandparent	1
legal guardian	1
Reason for contacting NaviCare/SoinsNavi^b^	
resources or services	11
care coordination	5
respite	3
funding	2
caregiver education and support	9

^a^ Some participants did not provide complete demographic information. ^b^ Participants often had more than one reason for contacting NaviCare/SoinsNavi.

The characteristics of the children/youth with complex care needs cared for by the participants varied (see Table 2). Over half were males between the ages of 2 and 11 years old with autism (under neurologic conditions in Table 2), although a large number of children also had a mental health issue. It should be noted that many of the children had more than one diagnosis.

**Table 2:** Characteristics of Child/Youth with CCN (N = 28)^a^.

	n

Gender of child/youth with CCN (n = 28)	
male	19
female	9
Age of child/youth with CCN (n = 28)	
<2 years old	2
2–5 years old	9
6–11 years old	10
12–17 years old	5
>17 years old	2
Condition(s) of child/youth with CCN (n = 44)^b^	
neurological	18
mental health	12
genetic abnormality	5
gastrointestinal	1
cardiac	3
autoimmune	2
renal	2
no diagnosis	1

^a^ Four caregivers used NaviCare/SoinsNavi for more than 1 child with CCNs. ^b^ All reported conditions were counted.

**Figure 1:**
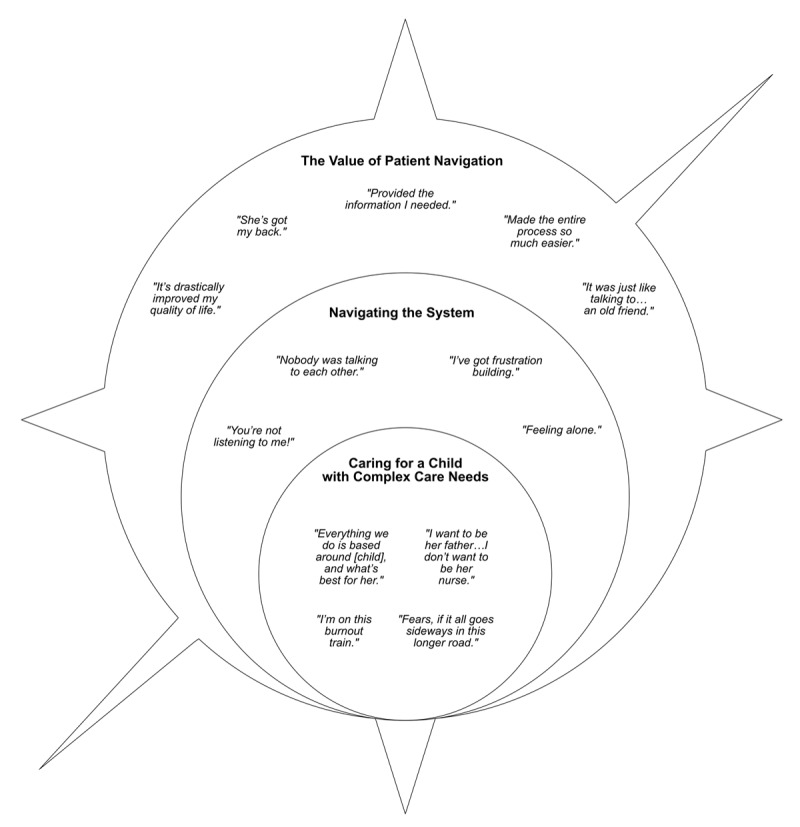
Main themes and sub-themes.

Three main themes emerged from the analysis of the data, including: 1) caring for a child or youth with complex care needs, 2) navigating the system, and 3) the value of patient navigation (see Figure 1). These themes were a result of mapping and merging thirteen sub-themes that arose related to the research questions. Each sub-theme is named using direct quotes (in vivo) to capture their overall meaning. Labels for main themes describe the content within each theme.

### Theme 1: Caring for a Child with Complex Care Needs

The first theme captures the experiences of caregivers caring for a child or youth with complex care needs. This theme not only provides context to enhance an understanding of these challenges, but it also highlights the fears, priorities, and concerns experienced by caregivers. These experiences are reflected in four sub-themes including: 1) “I want to be her parent…I don’t want to be her nurse”, 2) “I’m on this burnout train”, 3) “everything we do is based around [child], and what’s best for her”, and 4) fears, “if it all goes sideways in this longer road”.

#### “I want to be her parent…I don’t want to be her nurse”

Many caregivers expressed feeling challenged with providing the required ongoing physical care, medical care, and/or behavior management for their child or youth with complex care needs, which often led to a different relationship with their child or youth, as compared to their other children. This, coupled with the difficulty of getting someone to help with these needs due to the level of care required, cost, and availability, made it an ongoing challenge. As one caregiver explained:

“…there are very few people that we have come across that [could lift her]. I mean, my parents are in their 70s and we are in a rural community, so we can’t just go to a website and pull up people that are willing to come into our home or that we’re willing to let her go to their home”.

#### “I’m on this burnout train”

Parents also described caring for their child or youth with complex care needs as an “emotional rollercoaster”, leading to high levels of stress, fatigue, and burnout. One caregiver shared their struggles:

“So there’s a lot of anxiety and stress and worry and uncertainty…especially when they have a unique set of needs, and they’re going to require services beyond what maybe other children would require. The uncertainty is even greater, and it’s very daunting…you get really overwhelmed, because it’s a lot. So, I just break down and cry, and it’s just like there’s just no way I can do all this. I’m one person”.

#### “Everything we do is based around [child], and what’s best for her”

When caring for a child or youth with complex care needs, caregivers are in charge of coordinating appointments and trying to navigate multiple systems. This was described as time demanding and resulted in caregivers feeling as though their lives revolved around their child or youth. These demands often created barriers for working outside the home, and around their own self-care. As one caregiver expressed:

“it’s impacted my ability to work. It’s impacted my ability to do anything that isn’t my kid…100% of my life is dedicated to getting my son better and to making sure he’s got access to the right services”.

Another caregiver described it as “a life sentence”, stating it’s “an overwhelming feeling” and a “life-long ordeal”.

#### Fears, “if it all goes sideways in this longer road”

Caregivers shared their constant worries about their child or youth’s future. Specifically, they expressed concerns about their child or youth’s changing needs, as well as their transition to adulthood. This fear was explained by one caregiver:

“…the part that’s a little scary to me…we’re going to be backed into a corner and we’re going to be making decisions about her care that probably aren’t going to be the best. So if we could be proactive on the lookout for homes and find a cadillac for her over the next 2 or 3 years, I can live with that a lot better than I can live with blowing up and going sideways and not being able to provide the care…and then we’re backed into a corner and someone else takes over and throws her into a place that we have no say over. I mean, that’s a hard pill”.

Caregivers were also worried about not being able to take care of their child or youth as they themselves aged, as expressed by one caregiver, “I’m getting no younger, and it’s getting harder and harder”.

### Theme 2: Navigating the System

This theme describes caregivers’ perceptions and experiences of navigating the various programs and services needed for their child or youth with complex care needs. Four sub-themes illustrate the challenges and barriers caregivers faced while trying to navigate through the multiple systems involved in their child’s care, including: 1) “you’re not listening to me!”, 2) “nobody was talking to each other”, 3) “feeling alone”, and 4) “I’ve got frustration building”.

#### “You’re not listening to me!”

Many caregivers felt they were not listened to or respected, even though they were the “expert for their child” and knew “what it is that they need”. As one caregiver explained:

“I am an expert with my care for my daughter, and medical practitioners and everybody that’s involved with her needs to respect that because I deal with her every day. So it’s a little frustrating that way”.

Caregivers also reported that they had to work hard to be advocates for their child or youth’s needs, for example:

“The doctor still didn’t refer me because she didn’t think it was the thing to do, she referred me only because I insisted”.

#### “Nobody was talking to each other”

Often caregivers described a lack of collaboration and communication between those working within the system, with caregivers stating:

“everybody’s stuff was separate…nobody was looking at everything…everybody was just looking at their pieces and not all together”.

This lack of synergy often forced caregivers to explain their story “over and over and over again”. One caregiver explained how confusing this lack of collaboration and communication was, which led to mixed messages:

“So we were going to one doctor, our neurologist here, and they would say, ‘I think we should do this.’ Then we would go to another doctor at the IWK [Izaak Walton Killam Hospital for Children], who he referred us to, and she would have a different plan. She would say, ‘I don’t really know why he would think that medication, we should do this one.’ And then, we would come back. The two of them, even though they had referred, were not communicating, so we were getting conflicting information on what medication would be better. So at that point I was like, we don’t know which one to do. One person is saying, ‘No, I don’t think this is going to be helpful’, and the other one does!”

Caregivers also shared how they often feel as if their messages and inquiries just went into a “black hole” where you would not get a reply, or would have to wait weeks to hear back. As one caregiver described:

“She (child) needed help, she was really sick and having a lot of seizures, and I couldn’t get anyone to get back to me…we were just spinning our wheels”.

#### “Feeling alone”

Caregivers felt the onus fell on them as parents to “put all the pieces together” when caring for their child or youth. They described feeling overwhelmed, alone, and needing help, but not knowing where to start. They did not feel the system was compassionate to their needs, as articulated by one caregiver:

“You feel like you’re bleeding out in the backseat of a car and nobody is driving the car to the hospital. So you’re just sitting there, bleeding out, and there’s nobody there to help you”.

#### “I’ve got frustration building”

Caregivers described the various barriers and gaps in the system that made it hard to access care, which created a general feeling of frustration and bureaucracy. These included things such as the amount of paperwork needed to be evaluated for services, wait times to access services, lack of resources available, and restrictions on funding (e.g. did not qualify if income was too high, or if used up allotment). A lack of diagnosis also made working within the system more of a challenge. As one parent explained:

“It’s just so much paperwork and bureaucracy that we had to jump through by ourselves when we were going through this really stressful time that was taking up all of our attention”.

### Theme 3: The Value of Patient Navigation

This final theme describes the reasons caregivers called the centre as well as their experiences and satisfaction in working with NaviCare/SoinsNavi and the patient navigator. This theme is illustrated through five sub-themes including: 1) “she’s got my back”, 2) “made the entire process so much easier”, 3) “it was just like talking to…an old friend”, 4) “provided the information that I needed”, and 5) “it’s drastically improved my quality of life”.

#### “She’s got my back”

Caregivers shared they received ongoing support and encouragement from the patient navigator and valued knowing there was someone to reach out to when needed. Many caregivers expressed the follow-up was regular and extensive and felt the navigator “was looking out for” them. As expressed by one parent:

“I mean they were unbelievably supportive and encouraging. They understood where I was coming from, they were empathetic, they were like, ‘don’t worry, everything will be fine. Just take a breath and we will find what we can, and we will get back to you. And if we find all that stuff and if you get here and something’s not working, you call us back and we’ll work with you again’. It’s…we’re here until you literally don’t need us anymore, and that’s amazing”.

Some caregivers also felt that by having the patient navigator, they had an advocate for their child or youth’s needs and a voice in the system. As one caregiver shared, having the patient navigator added credibility and influence:

“It’s not just me talking to a receptionist, it’s a healthcare provider talking to another healthcare provider and I think that adds a little bit of leverage or weight to setting these appointments”.

#### “Made the entire process so much easier”

Most caregivers shared working with the patient navigator was helpful and easy, describing them as knowledgeable, well organized, and that they took the time to help find solutions. As one caregiver expressed:

“I’m extremely satisfied. I mean, having to navigate various systems to access supports and assistance, this was by far probably the easiest process I’ve had to go through”.

Many caregivers also felt the patient navigator helped ease the process through connecting, coordinating, and advocating on their behalf with other care providers to improve access to services. As one caregiver explained:

“She [patient navigator] put me directly in contact with the social worker. Basically got me all the forms I need to fill out to sign up… it was always one of those things that we had intended on but never actually did. Again, when I’m by myself, I work full-time, and to try to add that on top of it, it was just [too much]. So basically it was, ‘Here’s the forms, fill them out, send them back to me, and I’ll get them submitted, set up the interview’. She gave me the phone numbers… I believe she contacted them directly to see where we were with the whole interview process”.

Other caregivers felt the support received through NaviCare/SoinsNavi and the flexibility of communication methods (e.g. email, phone) “saved time”. For caregivers living in rural settings, the virtual nature of NaviCare/SoinsNavi was also more conducive to their needs. As one rural caregiver explained:

“everything was done over the phone or through email, which worked fine for me, just because of demographics of where I am”.

Some caregivers expressed they wished they found NaviCare/SoinsNavi in the earlier days of caring for their child or youth with complex care needs when they were trying to search for supports and resources, as articulated by one parent:

“if I had had NaviCare in my life far earlier, I think it would have made this road that we’ve had to journey down, I think it would have made it a whole lot easier”.

#### “It was just like talking to…an old friend”

Many caregivers shared “talking to somebody who’s not judging you” and feeling listened to was very helpful. They shared the communication was two-way and “participatory”, which provided a sense of validation, connection, and understanding. As one caregiver depicted their experience of talking with the patient navigator:

“She got it. It wasn’t like she was brushing me aside; it was like, she was listening and sometimes, you know, that’s all a person needs. Just to know that they’re being accounted for”.

Others shared that the patient navigator’s approach was person-centred and holistic toward them and their child or youth, as one parent explained:

“she [patient navigator] put a human element into what she was doing, so it was a very positive experience”.

#### “Provided the information that I needed”

Caregivers called for a variety of reasons including seeking: education materials so they could learn more about their child’s condition to better support them; referrals to funding opportunities for equipment and programs; and information on programs and services available in their communities. The participants reported that the centre provided coordination, navigation, and follow-up support with various services. In addition, the patient navigators not only directed them to appropriate services by providing them with lists, but they also assisted in the process of gaining access to those services. Many of the resources and services regularly sought after included help with physical care, respite, and mental health. As stated by one participant:

“She [patient navigator] helped us get into and in contact with Social Development, which we hadn’t before. And we now have programming set up…for respite care and we have a few items that they’re looking at, possibly another walker, as well as a stander”.

Many caregivers were looking for help and direction for what programs and services were available for their child or youth, or to confirm they were not missing opportunities for assistance (that they may not know about). As one parent shared:

“I wasn’t aware that Community Living had anything of value for someone in our situation. I didn’t know about them. And I didn’t know that there was any kind of a provincial support available. No-one had ever suggested either of those two things in all of my running around”.

Caregivers also reached out to the patient navigator to obtain assistance with processes and paperwork to navigate through the system. The patient navigator connected, coordinated, and advocated on behalf of the caregivers to other care providers to improve access to services. As one caregiver explained:

“She [patient navigator] put me directly in contact with the Social Worker. Basically got me all the forms I need to fill out to sign up… it was always one of those things that we had intended on but never actually did. Again, when I’m by myself, I work full-time, and to try to add that on top of it, it was just [too much]. So basically it was, ‘Here’s the forms, fill them out, send them back to me, and I’ll get them submitted, set up the interview’. She gave me the phone numbers…I believe she contacted them directly to see where we were with the whole interview process”.

Even caregivers that had been caring for a child or youth over a number of years and were familiar with the system, reached out to NaviCare/SoinsNavi to “to see if there was something else” they “didn’t know about”. One family just wanted to confirm “what we were looking for did not exist”. Finally, the support and information provided by the patient navigator helped families feel better prepared:

“The navigator was amazing. Like she kept in contact with me the whole time…she tried everything that she could. And the folder of information…my folder was full. It was stocked. She told me everything, like to put all my bills in there… she prepared me for this stuff”.

#### “It’s drastically improved my quality of life”

Many caregivers reported that working with the patient navigator, “lifted this huge weight” and took “the emotional anxiety and stress away” regarding getting help for their child or youth. The resources and services they were connected with through the navigator provided needed support, helped decrease their stress in caring for their child or youth, and improved their quality of life. Some also reported their ability to care for their child or youth improved through the support and resources arranged. As one caregiver shared:

“…it’s made so many things so much easier. I’m trying to think of a facet of my life which has not been impacted positively by this, and I really can’t. Like, in every way, every essence, just like waking up and going through a day, this piece has helped because it’s taken one less thing off my plate every day that was receiving a lot of attention”.

Another caregiver compared NaviCare/SoinsNavi to a light at the end of the tunnel, describing how it “massively” improved the quality of life “not just for myself, but for my kids”:

“…we had no resources, we had no idea where to turn, what to do. You pick up the phone and you call one person and then there’s a light. The light suddenly appears at the end of the tunnel, and you go, ‘Okay, if I can just get a little bit further, then all of this will happen’. And that’s been huge. Absolutely huge”.

## Discussion

Three broad themes emerged from the data: 1) experiences of caring for a child or youth with complex care needs; 2) trying to navigate various systems to meet the needs of their child or youth; as well as 3) motivations for seeking navigational support as well as the experiences and level of satisfaction working with a patient navigator. While the needs and experiences of caregivers are often overshadowed by the needs of their child or youth with complex care needs [12], their perspective is crucial to expanding our understanding of the circumstances and context within which the child or youth will be supported. The experiences shared by the caregivers in this study provide context to better understand caregiver experiences and satisfaction following their interaction with a patient navigation centre, as well as offer valuable insights into the daily struggles, changes in relationships, juggling of responsibilities, stresses, fears, and concerns these caregivers face when caring for a child or youth with complex care needs.

As shared by many of the caregivers, they often had to assume many additional roles when caring for their child or youth with complex care needs beyond being the primary caregiver, including being an advocate, care coordinator, and medical expert [13]. They also experienced challenges and frustrations when navigating the various systems, such as not being listened to, poor communication between care providers, and system barriers. Not only do these aforementioned experiences contribute to the negative impacts described above, they also suggest that the current system does not meet the needs of caregivers and adds to their burden. Caregivers of children or youth with complex care needs consistently report that they have unmet needs that exist beyond the immediate needs of their child or youth. Specifically, caregivers identify a need for emotional support, navigation, and information [12]. Furthermore, as demonstrated in the findings and in keeping with existing literature, being a caregiver to a child or youth with complex care needs can have a substantial impact on emotional and mental health [14,15], physical health [16], social connections [14], finances [17], and employment [18]. A holistic approach that includes caregivers is vital to the successful integration of care for children and youth with complex care needs as this can have a considerable impact on caregiver well-being, and the care they are able to provide to their child or youth with complex care needs [16].

The reasons for contacting a patient navigator in this study varied greatly between caregivers, further highlighting the diverse unmet needs of this population and existing gaps in the current systems. Many caregivers contacted NaviCare/SoinsNavi looking for information on disease states, medication, and funding; others were looking for programs and services or to validate they had not missed opportunities; while some needed assistance with processes and paperwork to navigate and improve access to programs and resources within the system. These requests are squarely aligned with the role of patient navigator, which has been described to include: care coordination and collaboration; support and information; advocacy on behalf of the patient; administrative activities; psychosocial support; assisting with access to services and resources; and reducing barriers to care [19]. As suggested by King et al. [12], the family-oriented services provided through a patient navigator can help to lessen the burden placed on the caregiver and enhance their ability to care for their child or youth. In this way, patient navigation contributes to the goal of integrated care and improves the caregiver experience.

According to Kodner and Spreeuwenberg [4], although integrated care must seek to improve alignment, and collaboration across sectors and settings, it must do so with the primary goals of improving quality of care, enriching one’s quality of life, increasing satisfaction, and enhancing efficiencies within the various systems. Our findings illustrate how patient navigation can positively impact these various factors to improve the integration of care for children and youth with complex care needs and their families. The experiences and stories shared during the interviews and captured in sub-themes such as, “she’s got my back”, “made the entire process so much easier”, and “it’s drastically improved my quality of life,” illuminate the value this type of service can offer to caregivers of children and youth with complex care needs. These findings suggest patient navigation services, such as those offered by NaviCare/SoinsNavi, can help caregivers with the daily “rollercoaster” of challenges caregivers experience when caring for a child or youth with complex care needs that have been documented in the literature [13,15]. This study also validates how patient navigation can enhance experiences in care through holistic support and building trusting relationships [20].

This paper has highlighted both the need for patient navigation, as well as the value it can provide to caregivers of children or youth with complex care needs. Although all participants contacted the centre seeking navigational support, this does not ensure that they would be satisfied with the support they received from the centre’s patient navigator. The theme that refers to the value of patient navigation emerged from the experiences of a number of participants who reiterated that navigation was a beneficial model of care. Future studies exploring the long-term outcomes of such patient navigation programs on the mental and physical health of caregivers, as well as the impact on social and vocational roles, would add to our understanding in this area. Additionally, evaluating different navigational models to determine what aspects are critical for success and for the development of operational standards would be of great value to ensure quality of care.

## Limitations

While the findings add valuable insights for NaviCare/SoinsNavi, and for the development and implementation of other new and existing patient navigation programs and services, the data collected was qualitative and limited to clients of this centre who agreed to participate in the study; therefore, they cannot be generalized to other patient navigation centres. To address this concern, we have shared details about the setting and intervention to help others who may be interested in assessing the transferability of this research to other contexts. Also, although all clients were asked to participate in the study, only those NaviCare/SoinsNavi clients who had time and interest volunteered to participate in the interviews. This could limit the breadth of the findings, as other clients who did not volunteer for a variety of reasons may have had different levels of satisfaction or experiences to share. The requirement for participants to self-report satisfaction and experiences about the services they had received from NaviCare/SoinsNavi was largely dependent on participant recall and recollection. The information shared by participants could also be limited by social desirability, where participants may not have wanted to say anything “bad” about the centre or may have unduly worried it may get back to the patient navigators, despite being informed that only the research team would have access to the findings. These limitations may have potentially affected the accuracy of details shared.

One of the challenges when conducting qualitative interviews is with recruitment. Although all clients who worked with a patient navigator with NaviCare/SoinsNavi were invited to participate in the study, it is possible that only those who had a more positive experience were inclined to participate. It can be challenging to ensure that both good and bad experiences are represented in the study sample. Also, it should also be noted that while most participants were well educated, almost a third had either some high school, completed high school, or had only completed some college or university. Future research could focus on recruiting clients from more priority neighborhoods and seeing if their experiences are similar to those reflected in this study.

## Conclusion

Caring for a child with complex care needs is challenging and can leave caregivers feeling overwhelmed, fearful, and alone. In addition to these burdens, caregivers also face a variety of challenges, gaps, and barriers in the system as they attempt to access the necessary programs and resources to better support them on this journey. The patient navigation services offered through NaviCare/SoinsNavi enhanced the overall caregiver experience of caring for a child or youth with complex care needs. Caregivers reported decreased levels of stress, feeling supported, and increased knowledge about health and social care systems. Through facilitating more convenient and integrated care, and improving access to education, supports and resources, patient navigation can be an innovative approach to support the needs of caregivers who have a child or youth with complex care needs. These findings can be used to inform research, practice, and policy on how to best improve care coordination, enhance patient experiences, and identify gaps and barriers to care.

## Author Information

All authors are affiliated with the centre. SD and AL are Co-Directors and KL is a Postdoctoral Fellow. SD secured the original funding that supported the needs assessment and development of the centre. AL was initially hired as a Postdoctoral Fellow with the goal to lead the evaluation of the centre and has since moved into a position of research associate at the university and Co-Director of NaviCare/SoinsNavi. Although all three authors are affiliated with the centre, we do not believe this has affected the quality of the research.
